# An Account of the Effects of Vitriolic Æther in a Case of Spasmodic Affection of the Stomach; and in Two Cases of Intermittent Fever

**Published:** 1794

**Authors:** William Davidson

**Affiliations:** Apothecary in London.


VII. *An Account of the Effects of Vitriolic Æther in a Caſe of ſpaſmodic Affection of the Stomach; and in two Caſes of Intermittent Fever. By Mr.* William Davidſon, *Apothecary in London.*

IN the month of November, 1791, a young married lady, of a delicate habit, having undergone much fatigue, was ſeized with a violent ſpaſm of the ſtomach, which came on immediately after dinner, every day, for eight days, and continued ſometimes for one, and ſometimes for two hours, without her finding any relief, although although a variety of warm things were given to her for that purpofe. On the 9th day fhe dined at my houfe, and was taken as ufual. Having a high opinion of æther as an antifpafmodic, I gave her a draught compofed of a drachm of vitriolic æther, and fimple peppermint water, defiring her to drink it quickly, which fhe did.

The moment it got into the ftomach it gave a confiderable fhock, (if I may fo exprefs myfelf) to the whole conftitution. She thought it very ftrong, and faid I had nearly fuffocated her; however, the pain went off immediately, and never returned after. *Quære*, was not this effect produced by the fhock given to the common fenforium through the medium of the ftomach, from which the diftribution of nervous influence was difturbed, and a ftop thereby put to the difeafed action then exifting?

Seeing fuch fuccefs from this medicine, and the commotion it excited in the body, I conceived it well adapted for anfwering one of the intentions expreffed by the illuftrious Whytt, in his Treatife on Nervous Difeafes; and I refolved to put it in practice as foon as an opportunity fhould offer, which happened foon after, as will appear by the following cafe of a quartan intermittent, which was ſpeedily ſtopped by it. The paſſage alluded to in Dr. Whytt's work, being of great importance towards eſtabliſhing a rational pathology of ſome nervous diſeaſes, I ſhall beg leave to mention it. He ſays, page 221, (8vo edition) "And as an intermittent "agrees with epileptic and other convulſive "diſorders, as to its cauſe, ſo its returning pa- "roxyſms, like theirs, may be often prevented "or weakened by raiſing, a ſhort time before "the approach of the fit, an acute pain or any "great commotion in the body." And the practice appears very rational with a view to diſturb or ſuſpend the nervous influence. The latter is to be moſt earneſtly wiſhed for in the treatment of theſe complaints: for, if ſuſpended only for a moment, there is great probability that the exiſting morbid action will either ceaſe or be changed into a milder affection. But if this cannot be accompliſhed, we often gain our purpoſe by diverting the nervous influence into a new channel.

Caſe

*Case of a Quartan Intermittent cured by Æther.*

Mrs. K—, aged about forty-six years, having lost her only child on the 30th of August, 1791, was so much affected, that she became very low and weak; and, towards the end of September, was seized with a severe quartan ague, which continued to return at regular periods, but for which she used no remedy until the 15th of January, 1792, when she applied to me. She was then in a feeble state, but had no particular symptom of visceral obstruction.

Having first cleared the *primæ viæ* by an emetic and a purgative medicine, I began to treat her in the usual way, by giving the bark and different tonics in the intervals of the fits, and endeavouring to shorten the paroxysms, when present, by the common methods.

This plan of treatment I pursued until the 5th of February, without any benefit as to the periodical returns, although her general health was better. Being now tired and disappointed, she would take no more medicine; and in this resolution she persisted until the 16th, when she again ſolicited my aſſiſtance. She was now extremely weak and thin, and her ague had regularly continued.

Having been unſucceſsful in my former attempts, I reſolved to adopt Dr. Whytt's method of exciting a commotion in the ſyſtem juſt before the approach of the fit. With this view I ordered an æther draught, ſimilar to that given in the above caſe of ſpaſm, to be taken when the leaſt feeling of the fit ſhould come on. Some minutes, however, elapſed after the acceſſion of the cold fit before the draught was taken; which, notwithſtanding, produced the deſired effect. When taken into the ſtomach it gave her ſo conſiderable a ſhock, that ſhe imagined ſome miſtake had been made in preparing her medicines, and was therefore much frightened. The coldneſs went off immediately, and being ſucceeded by a pleaſant ſenſation of warmth, without any fever or perſpiration, ſhe was in good ſpirits all the reſt of the evening.

Fearing this one doſe might not be ſufficient, as the fit had commenced before the draught was taken, I requeſted ſhe would take another at the time the next paroxyſm was expected, whether it appeared or not. Accordingly, on the the 20th, perceiving (to ufe her own expreffion) her nails were becoming black, which fhe had found to be the conftant precurrent fymptom of a fit, fhe took her draught, which affected her ftill more than the former.

At firft fhe feemed like to burft, (as fhe expreffed herfelf) but foon after felt comfortable, and fpent a very cheerful evening. Her ague difappeared from this time, nor has fhe had any return of it fince. She took fome tonic medicine twice a day for feveral days, and acquired frefh ftrength and fpirits daily.

In the above cafe it is probable that no particular vifceral obftruction exifted, and that the difeafe originated from weaknefs of the nervous fyftem, brought on by grief for the lofs of her child; and might therefore be confidered as a nervous difeafe that had become periodical, and as fuch was to be treated like epilepfy, and fome others of that clafs; many of which might be fuccefsfully attacked on the above principle. That many intermittents are nervous, would appear probable from the great number of patients who are daily cured by frights, charms, accidents, or, in fhort, any thing which, for the moment, diverts or fufpends the nervous influence, and thus breaks the habit of attack.

In

In confirmation of this idea, I ſhall take the liberty to add a few more facts. A worthy baronet, whom I have the honour to attend, having been affected with an obſtinate ague for eighteen or twenty months, for which he had tried the bark, given in the beſt manner, without effect, was determined to follow the hounds on the approach of the next fit, in hopes that the exerciſe might prevent it from coming on.

He made the attempt, and certainly ſucceeded; for in leaping a five-barred gate, his horſe fell, by which accident he broke one of his collar bones, and his ague never appeared after. Here the violent ſhock to the conſtitution from the fall, and the ſymptomatic fever induced by the fracture, cured the intermittent; perhaps by ſtopping the habitual diſeaſed action, and inducing another action, where the nervous influence was determined more firmly and ſteadily to a particular point.

Many patients have been cured by falling accidentally into a river on the acceſſion of the cold fit. A large doſe of laudanum has alſo ſtopped a fit; and a ſimilar effect has been produced by a glaſs of brandy and hartſhorn. I know a very reſpectable private gentleman, who has cured many agues by the following draught, of his own compoſing; viz.

viz. brandy, ſtrong vinegar, and water, of each, a wine-glaſs full, to be taken juſt before the fit. Every practitioner is more or leſs acquainted with facts of this kind ; and however mortifying it may be for profeſſional men to hear, yet it is alſo well known that an ague, which has for months baffled the united efforts of talents and experience, has been often cured by ſome charm, or by great emotion of mind, excited by the ſagacity of ſome good old lady on the approach of the fit. That the principle is rational, and conſiſtent with the laws of the animal œconomy, will appear probable from obſerving the rules of the nervous influence, which cannot be directed to two points, at one and the ſame time, with any degree of ſteadineſs or permanency.

*Caſe of a Tertian Intermittent ſtopped by Æther.*

Towards the end of February, 1793, a young man, about twenty-four years of age, of a delicate ſtructure, and fair complexion, became feveriſh, and was perſuaded by a medical friend to be blooded. Immediately after the operation was performed, he grew weak, hyſterical, and continued in a fainting ſtate, more or leſs, during the remaining part of of the day. The next morning, about eight o'clock, an intermittent, of the tertian type, made its appearance, and went through the ufual ftages. After proper attention had been paid to the ftate of the firft paffages, the bark was given every two hours in the abfence of the fit. This plan, with the addition of a nourifhing diet, and the occafional ufe of port wine and porter, was continued for four days, but with no good effect; and the cafe of Mrs. K— having occurred to my remembrance, I now thought of trying the fame means of cure in this cafe.

Accordingly, on the 8th of March, I fent him the fame æther draught, as had been given in the former cafes, to be taken on the approach of the next paroxyfm, which he expected the following morning. On the 9th he took his draught, exactly as the paroxyfm was beginning, and it went off directly by a gentle moifture on the fkin. Having proceeded with his bark for fix days, gradually diminifhing the frequency of exhibition, and having had no return of his ague, I left him feemingly in perfect health.

From the above cafes it appears evident, that fome intermittents may be cured by exciting the the commotion recommended by the celebrated author above mentioned. It remains, therefore, with practitioners to ascertain what is the best medicine for answering this indication: Whether æther, electricity, a shock from the cold bath, or any particular impression on the mind, is the best; or whether they ought not all to be adopted occasionally, and only varied according to the different constitutions affected, and the different states of each different patient; and also, whether all kinds of agues can be cured or relieved in this way. I exhibited the æther twice to a patient labouring under an intermittent, who was much affected with different internal complaints, and where the kidnies did not perform their functions, without stopping the disease, although the fit was much shortened by the second dose. From this I suspect that the method above practised will not succeed when the disease is attended with visceral obstructions; but that when the ague arises from debility, occasioned by grief, or whatever weakens the nervous system, from which habitual fits have been induced, the habit of attack may be stopped, and the disease cured as already mentioned.

But I presume the success, even in the latter cases, cafes, will depend much upon the commotion being excited as near as poffible to the moment of attack. I have lately fucceeded in one cafe of epilepfy, which was of many years ftanding, and had been induced by intenfe application of mind, by giving an æther draught on the approach of the fit, and purfuing a tonic plan in the intervals.

